# A study of critically ill obstetric patients admitted to intensive care unit of a tertiary care hospital

**DOI:** 10.12669/pjms.40.7.7734

**Published:** 2024-08

**Authors:** Wajeeha Syed, Nazia Liaqat, Muhammad Shehryar Ashraf, Nayab Khan

**Affiliations:** 1 Wajeeha Syed, FCPS, MRCOG Associate Professor, Department of OBGYN, Medical Teaching Institute, Lady Reading Hospital, Peshawar, Pakistan; 2 Nazia Liaqat, FCPS Associate Professor, Department of OBGYN, Medical Teaching Institute, Lady Reading Hospital, Peshawar, Pakistan; 3 Muhammad Shehryar Ashraf, MCPS, FCPS, CICM Assistant Professor, Department of Anesthesia and Critical Care, Medical Teaching Institute, Lady Reading Hospital, Peshawar, Pakistan; 4 Nayab Khan, FCPS, Department of OBGYN, Medical Teaching Institute, Lady Reading Hospital, Peshawar, Pakistan

**Keywords:** Acute renal failure, Critically ill obstetric patients, DIC, Outcome

## Abstract

**Objectives:**

To evaluate characteristics, indications, complications and outcome of obstetric patients admitted to ICU of tertiary care hospital in KPK, Pakistan.

**Methods:**

This descriptive study was conducted in department of OBGYN of Lady Reading Hospital, Peshawar from January 2021 till December 2021. A total of 62 patients were enrolled into the study using nonprobability consecutive sampling technique. Their data were collected on a proforma. All patients were followed till their death or discharge home from hospital.

**Results:**

The mean duration of ICU stay of patients, was 6.85 ± 4.82 days. Out of 62 patients 17 (27.41%) expired in ICU, while 45 (72.58%) patients survived and were discharged. Pre-eclampsia and Eclampsia was the commonest primary diagnosis, accounting for 28 cases (45.2%) with a case fatality rate of 25%, followed by 13 cases (21%) of primary postpartum hemorrhage (PPH) as the second commonest reason for ICU admission and a case fatality rate of 38%. The underlying primary diagnosis had no statistically significant association with outcome of the patient. Acute Renal failure had statistically significant association with outcome of the patient with adjusted OR 4.79, CI:1.17-19.66, p-0.02. Similar positive association with mortality existed for patients having DIC (aOR:6.59; CI:1.34-32.34, p-0.02).

**Conclusion:**

Pre-eclampsia/Eclampsia is the commonest reason for intensive care admission, however PPH has the highest case fatality rate. The outcome of critically ill obstetric patients is dependent on complications and not primary underlying diagnosis.

## INTRODUCTION

ICU admission is the most sensitive and critical part of care provided to a patient especially when it is about rescuing life of a mother. It is basically a clinical entity which provides organ support including mechanical ventilation to needy patients.^1^ Treatment in ICU is a continuum of care provided to a parturient woman who is facing adverse outcomes of pregnancy. As antenatal population is mostly young and healthy, their risk of having complications is remote.^2^ However exceptions are always there. Rarely women can land up in an intricate state due to preexisting medical comorbidities or due to conditions specific to pregnancy like preeclampsia and postpartum hemorrhage.^3^

In poor countries like Pakistan illiteracy, poverty, lack of women empowerment as general primary factors and nutritional deficiencies, anemia, poor antenatal care, mishandling by midwives, late hospital referrals as secondary factors, add to the complexities a mother can face during her course of pregnancy.^4^ According to a report published in Daily Times nearly 8.6 million women become pregnant in Pakistan and of these 1.2 million are likely to face obstetric complications putting them at risk of ICU admission.^5^

Critically ill obstetric patients account for as much as 7% of ICU admission in developing countries while they account for only 0.2 % to 0.9% in developed countries.^6^,^7^ Better health care facilities and easy access to specialized obstetric service account for this difference.^8^ The objective of this study was to evaluate characteristics, indications, complications and outcome of obstetric patients admitted to ICU of tertiary care hospital in KPK, Pakistan. The result of the study will help us to analyze our services, identify deficient areas and hence improve the care for needy patients.

## METHODS

This descriptive case series study was conducted in department of OBGYN at Lady Reading Hospital from January 2021 till December 2021.

Sample size of 62 was calculated via OpenEpi online calculator, by taking confidence level as 95%, confidence limit as 5% and mortality proportion of obstetric ICU patients as 8%.^9^ Sampling was done by consecutive non probability method. All patients whether antenatal at any gestation or within 42 days of delivery requiring admission in ICU were enrolled in the study. Consent was obtained by next of kin if patient herself could not sign it. Information regarding maternal age, parity, antenatal, postnatal status, mode of delivery, primary diagnosis, complications, hospital stay, death or survival was recorded in a predesigned proforma. SPSS version 23 was used to analyze the data. Frequencies and percentages were calculated for categorical variables and chi square test was applied to determine associations. For significant associations logistic regression analysis was done to determine strength of associations and to control for confounders. Mean and standard deviations were calculated for quantitative variables.

### Ethical Approval:

It was taken from ethical committee of the hospital (ERB Ref # 253/LRH/MTI, dated :20-10-2021).

## RESULTS

The mean age of sample of 62 patients was 27.82±6.01. Mean gestational at the time of delivery was 35.67±4.54, whereas mean duration of ICU stay was 6.85±4.82 day. Vaginal birth was observed in 23(37.06%) while cesarean delivery was present in 20 (32.25%) patients. Cesarean hysterectomy was done in 8(12.9%), postpartum hysterectomy in 9 (14.5%) and laparotomy in 2(3.22%) cases. Mean gestational age at delivery did not show statistically significant association with outcome of patient (Mean difference 1.5; CI:0.5-4.09, p-0.243). Surgical interventions were observed to have no association with outcome of the patients. (p >0.05).

**Fig.1:**
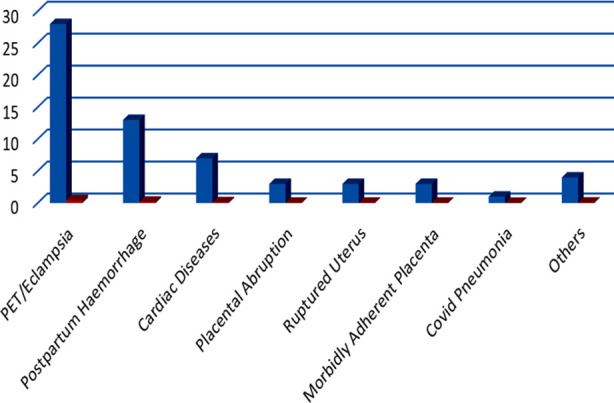
Primary Diagnosis of patients referred to ICU.

Pre-eclampsia and Eclampsia was the most common primary diagnosis, accounting for 28 cases (45.2%) with a case fatality rate of 25%, followed by 13 cases (21%) of primary postpartum hemorrhage as the second commonest reason for ICU admission and a case fatality rate of 38%. There were seven cases (11.3%) of cardiac diseases. These cardiac conditions included severe rheumatic heart disease with pulmonary artery hypertension in three cases, peripartum cardiomyopathy in two cases and two cases of congenital cardiac diseases. There were three cases each of placental abruption, morbidly adherent placenta and ruptured uterus, (Fig.1). Out of 62 patients 17 (27.41%) expired in ICU, while 45 (72.58%) patients survived and were discharged.

The underlying primary diagnosis had no statistically significant association with outcome of the patient. (Table-I). Out of 17 cases who expired, 15(88.2%) had two or more than two system complications while two cases had only one system complication (p-0.001). Sepsis complicated 22 (35.48%) cases. The association of sepsis with outcome of patients was not statistically significant. (p >0.05)

**Table-I:** Association of primary diagnosis to outcome of patients.

Primary Diagnosis	EXPIRED		Survived		Total	p-value

	N	%ages	N	%ages		
PET/Eclampsia	7	25 %	21	75	28 (100%)	0.91
Postpartum Hemorrhage	5	38.4%	8	61.5	13(100%)	0.51
Cardiac Diseases	3	42.8%	4	57.1	7(100%)	0.38
Placental Abruption	0	0%	3	100	3(100%)	0.55
Morbidly Adherent Placenta	2	66.6%	1	33.3	3(100%)	0.18
Ruptured Uterus	0	0%	3	100	3(100%)	0.55
Covid Pneumonia	0	0%	1	100	1(100%)	1.00
Others	0	0%	4	100	4(100%)	0.56
Row Total	17		45		62`	

Acute Renal failure and DIC has statistically significant association with outcome of the patient. This association was observed to persist on multivariate logistic regression. The adjusted odd ratio for association of acute renal failure and mortality was 4.79 (CI:1.17-19.66, p-0.02). Similar positive association with mortality existed for patients having DIC (p-0.02; aOR:6.59; CI:1.34-32.34), (Table-II).

**Table-II:** Association of complications to outcome of patients.

Complications	Expired	Survived	Total cases	p-value	Adjusted Odd Ratio	95% Confidence Interval
Acute Renal Failure	13	16	29	0.02	4.79	1.17-19.62
HELLP	04	11	15	0.22	0.38	0.07-1.79
DIC	10	12	22	0.02	6.59	1.34-32.35
Neurological complications	04	05	09	0.13	4.99	0.61-40.84
Cardiovascular complications	05	10	15	0.498	1.88	0.30-11.66
Sepsis	07	15	22	0.588	1.525	0.33-7.01

## DISCUSSION

The study depicted eclampsia, primary postpartum hemorrhage and cardiac diseases as three leading causes of ICU admission in obstetric patients. The outcome of patients was seen to be affected by the number and type of system complications. Almost half of the ICU admissions were due to eclampsia while a little less than quarter were due to postpartum hemorrhage. International studies statistics also mention above two as main reason for intensive care admission.^10^ However statistics of a South Nigerian hospital revealed that majority of ICU admissions were due to rupture uterus.^11^ Study in Aga Khan university hospital Karachi, Pakistan quoted figure of ICU admission due to postpartum hemorrhage as 21% and hypertension as 18.6%.^3^ Seven out of total sixty-two (11%) landed in ICU because of cardiac disease.

This figure is close to 13% quoted by Tsaitlin-Mor L et al.^12^ while in study by Qureshi et al.^3^ 7.2% had ICU admissions all due to peripartum cardiomyopathy. Cardiac diseases were the cause of admission to ICU in 12% cases in a study conducted in China.^13^ Improvement in medical technology has increased the survival of patients with congenital heart diseases. However, the physiological burden of pregnancy can unmask the compromised heart function in certain cases especially if they have not received proper preconception and antenatal care.

Increasing rate of repeat cesarean section and the resultant morbidly adherent placenta is one of the major causes of massive obstetric hemorrhage.^14^ Three patients in current study had ICU ingress because of excessive blood loss due to morbidly adherent placenta. Bailit JL et al. reviewed 158 cases of MAP in study spanned over three years and reported 39% admission rate to ICU in such cases.^15^ Qureshi et al. also concluded that MAP accounted for one third of ICU admissions because of extensive blood loss.^3^ In a Chinese study by Zhang M et al.^13^ 16 cases of ICU admission were due to placenta increta. Their study was over four years and analyzed one hundred and thirty three patients needing ICU admission.

Mishandling by untrained birth attendants resulting in obstructed labor, ruptured uterus, sepsis, organ damage and maternal morbidity and mortality is a bitter fact in developing world and Pakistan is no different. Ismail S et al.^8^ reported admission of two patients in ICU with rupture uterus. Both of them survived. In current review three patients of rupture uterus had ICU stay and fortunately all three survived. Placental abruption due to any cause can have lifelong damaging effects on maternal organs.^3^ Patients in this study were admitted in ICU as they had abruption and all were discharged after treatment in a healthy status.

The current review revealed that mortality was higher in patients who ended in acute renal failure and disseminated intravascular coagulation as a result of obstetric misfortune as compared to other complications. In study by Qureshi R et al.^3^ mortality was five times higher in patients with gastrointestinal complications. Adeniran AS et al. in their research article deduced that mortality was 100% among those admitted for amniotic fluid embolism, complications of unsafe abortion and peripartum cardiomyopathy.^16^

Mean maternal age was 27 years in the study. It was reported as 28 by Adeniran AS et al.^16^, in Cavazos Rehg review maternal age above 35 has been observed to be associated with maternal deaths after ICU admission.^17^ Khaskheli M et al. in their analysis quoted that 80% of mothers in ICU are between 20 and 40 years of age.^18^ Mean maternal age was 27.4 years in a study in Ghana where researchers found that woman above 35 years have twice the risk of death and those in age group 25 to 35 years have 67% increased risk.^10^

### Limitation of the study

This is a study of only 62 patients admitted in ICU in one year in Lady Reading Hospital and does not represent the status of all parturient in need of ICU admission in the region. The current study is one of the few studies from this region of the world, on this very important subject. However, this was a study without a control group and of small sample size.

## CONCLUSION

All studies of the same theme from developing countries point towards the need of improving health care system at primary and secondary levels so antenatal patients with risk factors are picked and referred in time to tertiary care. Because managing complications consumes more resources in comparison to preventive strategies.

### Authors Contributions:

**WS:** Conceived, designed, and prepared manuscript.

**NL:** Did statistical analysis, reviewed, and finalized manuscript.

**MSA:** Collected data and edited manuscript.

**NK:** Did literature search, data collection, and data management.

**WS** and **NL:** Take the responsibility and are accountable for all aspects of the work in ensuring that questions related to the accuracy and integrity of any part of the work are appropriately investigated and resolved.

## References

[1] Hashmi M, Taqi A, Memon MI, Ali SM, Khaskheli S, Shehryar M (2020). A national survey of critical care services in hospitals accredited for training in a lower-middle income country:Pakistan. J Crit Care.

[2] Heinonen S, Tyrväinen E, Saarikoski S, Ruokonen E (2002). Need for maternal critical care in obstetrics. A population-based analysis. Int J Obstet Anesth.

[3] Qureshi R, Irfan AS, Raza A, Khurshid A, Chisti U (2016). Obstetric patients in intensive care unit. JRSM Open.

[4] Aziz A, Saleem S, Nolen TL (2020). Why are Pakistani maternal, fetal and newborn outcomes so poor compared to other low- and middle-income countries?. Reprod Health.

[5] Qaise M Pakistan's maternal death shame. Daily times. Commentary/Insight. Oct. 22, 2019.

[6] Patnaik T, Samal S, Behuria S (2015). Obstetric admissions to the intensive care unit. A five-year review. Int J Reprod Contracept Obstet Gynecol.

[7] Sukhwinder KB, Sukhminder JSB (2012). Delivering Obstetrical critical care in developing nations. Int J Crit Illn Inj Sci.

[8] Ismail S, Sohaib (2019). Obstetric patients requiring critical care. Retrospective study in a tertiary care institute of Pakistan. J Obstet Anaesth Crit care.

[9] Bhadade R, De Souza R, More A, Hardo M (2012). Maternal outcomes in critically ill obstetric patients. A unique challenge. Indian J Crit Care Med.

[10] Anane FB, Agbano EK, Osarfo J, Opoku ADA, Boateng AS, Ken AS (2021). A ten-year review of indications and outcomes of obstetric admissions to an intensive care unit in a low resource country. PloS ONE.

[11] Ozumba BC, Ajah LO, Obi VO, Umeh UA, Enebe JT, Obioha KC (2018). Pattern and outcome of obstetric admissions into the intensive care unit of a Southeast Nigerian Hospital. Indian J Crit Care Med.

[12] Tsaitlin-Mor L, Nir A, Elchalal U, Bdolah-Abram T, Weiniger CF (2022). Outcomes of pregnancy and delivery in women with cardiac disease compared to matched healthy controls. J Matern Fetal Neonatal Med.

[13] Zhang M, Tang ZH, Wen HC, Wu YL, Gao XX (2021). Clinical characteristics and outcomes of obstetric patients requiring ICU admission:a 5 years retrospective review. Clin Exp Obstet Gynecol.

[14] Cheng KK, Lee MM (2015). Rising incidence of morbidly adherent placenta and its association with previous caesarean section:a 15-years analysis in a tertiary hospital in Hong Kong. Hong Kong Med J.

[15] Bailit JL, Grobman WA, Rice MM, Reddy UM, Wapner RJ, Varner MW (2015). Morbidly adherent placenta treatments and outcomes. Obstet Gynecol.

[16] Adeniran AS, Bolaji BO, Fawole AA, Oyedepo OO (2015). Predictors of maternal mortality among critically ill obstetric patients. Malawi Med J.

[17] Cavazos-Rehg PA, Krauss MJ, Spitznagel EL, Bommarito K, Madden T, Olsen MA (2015). Maternal age and risk of labor and delivery complications. Maternal Child Health J.

[18] Khaskheli M, Baloch S, Sheeba A (2014). Iatrogenic risks and maternal health:Issues and outcomes. Pak J Med Sci.

